# Variability and reproducibility in deep learning for medical image segmentation

**DOI:** 10.1038/s41598-020-69920-0

**Published:** 2020-08-13

**Authors:** Félix Renard, Soulaimane Guedria, Noel De Palma, Nicolas Vuillerme

**Affiliations:** 1 Univ. Grenoble Alpes, CNRS, Grenoble INP, LIG, 38000 Grenoble, France; 2 Univ. Grenoble Alpes, AGEIS, 38000 Grenoble, France; 3 Institut Universitaire de France, Paris, France

**Keywords:** Computer science, Medical research

## Abstract

Medical image segmentation is an important tool for current clinical applications. It is the backbone of numerous clinical diagnosis methods, oncological treatments and computer-integrated surgeries. A new class of machine learning algorithm, deep learning algorithms, outperforms the results of classical segmentation in terms of accuracy. However, these techniques are complex and can have a high range of variability, calling the reproducibility of the results into question. In this article, through a literature review, we propose an original overview of the sources of variability to better understand the challenges and issues of reproducibility related to deep learning for medical image segmentation. Finally, we propose 3 main recommendations to address these potential issues: (1) an adequate description of the framework of deep learning, (2) a suitable analysis of the different sources of variability in the framework of deep learning, and (3) an efficient system for evaluating the segmentation results.

## Introduction

Medical imaging plays a central role in medicine today because it can reveal the anatomy of the patient. However, to leverage the full potential of medical images, it is necessary to analyze them via image processing. One of the main clinical tools is image segmentation^1, 2^. Medical image segmentation can be defined as an automatic (or semiautomatic) process to detect boundaries within a 2D or 3D image. It is based on information such as pixel intensity, texture and anatomical knowledge.The result of segmentation can then be used in further applications and in gaining insights^2^; examples include the quantification of tissue volumes^3, 4^, diagnosis^5, 6^, the localization of pathology^7, 8^, the study of anatomical structure^9, 10^, treatment planning^11^, and computer-integrated surgery^12^.

Manual medical image segmentation leads to two main issues: much time is needed for delineation, and reproducibility is called into question. First, the time needed to segment is incompressible, and it is correlated with the number and the size of images. Since the size of these two parameters is increasing due to the ease of facility access to medical imaging and the improvement of acquisition technologies, manual segmentation is becoming intractable. Second, reproducibility corresponds to the agreement between the results of multiple measurements of the data (here, the segmentation results) under the same methodology. In medical image segmentation, it is well known that there is inter- and intraoperator variability. The former relates to the observed differences in the segmentation results obtained by two different operators, while the latter relates to the observed differences between two results of segmentation tasks performed by the same operator at two different times. Due to the crucial role of segmentation in medical diagnostics and treatments, the reproducibility of the method is fundamentally important.

These two issues lead one to consider automatic segmentation. Automatic segmentation consists in determining a prediction model and its inherent parameters relative to a given class of problems (for example, the kind of imaging performed or organs imaged). These parameters can be divided into two classes: the hyperparameters associated with the model and the parameters estimated from the dataset. The aim of automatic segmentation is to estimate the best parameters to obtain highly accurate results over the training dataset while maintaining good generalization for other datasets of the same class of problem, also called “test datasets”. In other words, the algorithm must avoid perfectly fitting the training set with poor accuracy results on the testing set. This problematic phenomenon is also called “overfitting” (see page 108 of the book^13^).

The rapid development of new automatic segmentation algorithms since the 2000s is strongly connected to the rise of machine learning^2^. During the last decade, a specific field of machine learning and artificial neural networks, called “deep learning” (DL)^13, 14^, has outperformed classical segmentation methods^15^. A neural network with several hidden layers is considered a ‘deep’ neural network, hence the term ‘deep learning’^14^. This is the case for several reasons, for example, nonsupervised feature extraction via convolutional layers and the possibility of dealing with a very large dataset via efficient optimization methods such as backpropagation of the gradient (see chapter 6.5 of the book by Goodfellow et al.^13^). Several DL architectures have been applied to medical image segmentation, including fully convolutional networks (FCNs)^16^ and U-Net^17^ (see Litjens et al.^15^ for a recent review). FCNs^16^ are built from locally connected layers, such as convolution, pooling and upsampling layers. An FCN is composed of two main parts: the downsampling and upsampling paths. The downsampling path captures contextual information, whereas the upsampling path recovers spatial information. Moreover, skip connections between layers are performed to recover fine-grained spatial information that is potentially lost in the pooling and downsampling layers. U-Net^17^ is built upon FCNs. The main difference is that each downsampling scale is linked to the corresponding upsampling scale with a concatenation operator. In this way, each upsampling scale has the information of the corresponding downsampling scale and the lower upsampling scale, leading to better segmentation.

However, although DL algorithms perform well, they are complex. A number of factors may explain the variability in the obtained results: the intrinsic variability of the dataset, the stochastic process during optimization, the choice of the hyperparameters relative to the optimization and regularization processes, and the choice of the DL architecture itself. This variability in the different parts of the framework leads to some difficulties in analyzing the reproducibility and making comparisons between frameworks. In addition, this variability leads to numerous parameters and hyperparameters being set. Furthermore, as highlighted in Joelle Pineau’s reproducibility checklist^18^, provided during NeurIPS 2019, describing the DL methods becomes its own challenge for reproducibility. Moreover, the strategy for evaluating the segmentation results, and thereby the variability of the method, is complex. There are a plethora of metrics^19^ to analyze segmentations, leading to various ways of comparing the methods.

Along these lines, three main questions, at least, about variability and reproducibility can be formulated.

- Question 1: Is there enough information in published articles in the field of medical image segmentation with DL to correctly reproduce the results?
- Question 2: If the information is provided, has the variability in the several steps of the DL framework been considered?
- Question 3: Does the evaluation system for the segmentation results correctly reflect this variability?

These three questions are crucial for the application and potentially the evaluation of the segmentation algorithms. After focusing on the concept of reproducibility in medical image segmentation and on how to consider the different sources of variability in DL, we will review the literature to provide an overview of the practice of reproducibility in the fields of medical image segmentation in DL, based on three main topics: (1) the description of the methods, (2) the analysis of variability and (3) the evaluation system. On the basis of this synthesis, we will propose recommendations to appreciate the results of new DL strategies.

## Related work

In this section, we will broadly address the issues of the reproducibility and evaluation of segmentation in medical imaging. Then, we will outline several sources of variability in the DL framework that can lead to difficulties for reproducibility.

### Reproducibility and evaluation of segmentation in medical imaging

Reproducibility is a popular topic in science^20^. Hence, numerous articles^21, 22^ reveal a potential *crisis of reproducibility* in the different fields of science. Thus, most scientists have experienced a failure to reproduce results^21^ (more than 50% in the case of their own works in medicine, physics and engineering and more than 75% in the case of works by another person in the same fields).

In the rest of the article, we will follow the definition of the report of the National Academies of Science, Engineering, and Medicine^20^: *reproducibility means obtaining consistent results using the same input data, computational steps, methods, and conditions of analysis; it is synonymous with computational reproducibility.* Moreover, this report^20^ (recommendation 5-1, page 7) recommends that *researchers should provide an accurate and appropriate characterization of relevant uncertainties when they report or publish their research.* These uncertainties include stochastic uncertainties.

Reproducibility can be assessed with different procedures. First, reproducibility can be analyzed by intraclass correlation (ICC), proposed by Shrout and Fleiss^23^. The score obtained, which is between 0 and 1, indicating poor and perfect reproducibility, respectively, enables a comparison between intra-individual and inter-individual variabilities. Another statistical tool generalizing the ICC is analysis of the variance^24^ (ANOVA). It provides a collection of tools focusing on the variability of the means among groups. One interesting point is that ANOVA can deal with multiple factors.

One of the main sources of variability in machine learning originates from the difference between the observed samples of the dataset and the real distribution of the dataset. The fact that the learning step of the algorithm is performed on only a part of the distribution can affect the reproducibility and particularly the replication of the results. A class of tools, called “cross-validation” (CV)^25^, is available in studying this variability. A special focus on these methods is made in the next section, concerning variability in a dataset.

Moreover, segmentation in the specific field of medical imaging is complex in terms of reproducibility for several reasons. First, the available datasets are generally limited: the number of samples is generally less than 100 items. Then, each segmentation task must be considered with regard to the image modality (for example, whether it was obtained by MRI, scanner, or echography) and the organ studied^26^. Furthermore, the masks in segmentation are usually generated manually. This leads to some intra- and inter-rater variability. Consequently, there is no certain truth but only a gold standard. Additionally, there are also several metrics to evaluate segmentation, such as the dice coefficient (DC) and the modified Hausdorff distance. Each metric focuses on a specific aspect of the segmentation^19^. For example, a metric can correctly reflect the good overlap between a segmentation mask and a gold standard, but it cannot highlight the smoothness of the contour. To correctly describe the quality of a segmentation, several metrics are necessary^19, 26^. An adequate system of evaluation will permit accurate consideration of the variability in DL frameworks.

### Variability in DL frameworks

**Figure 1:**
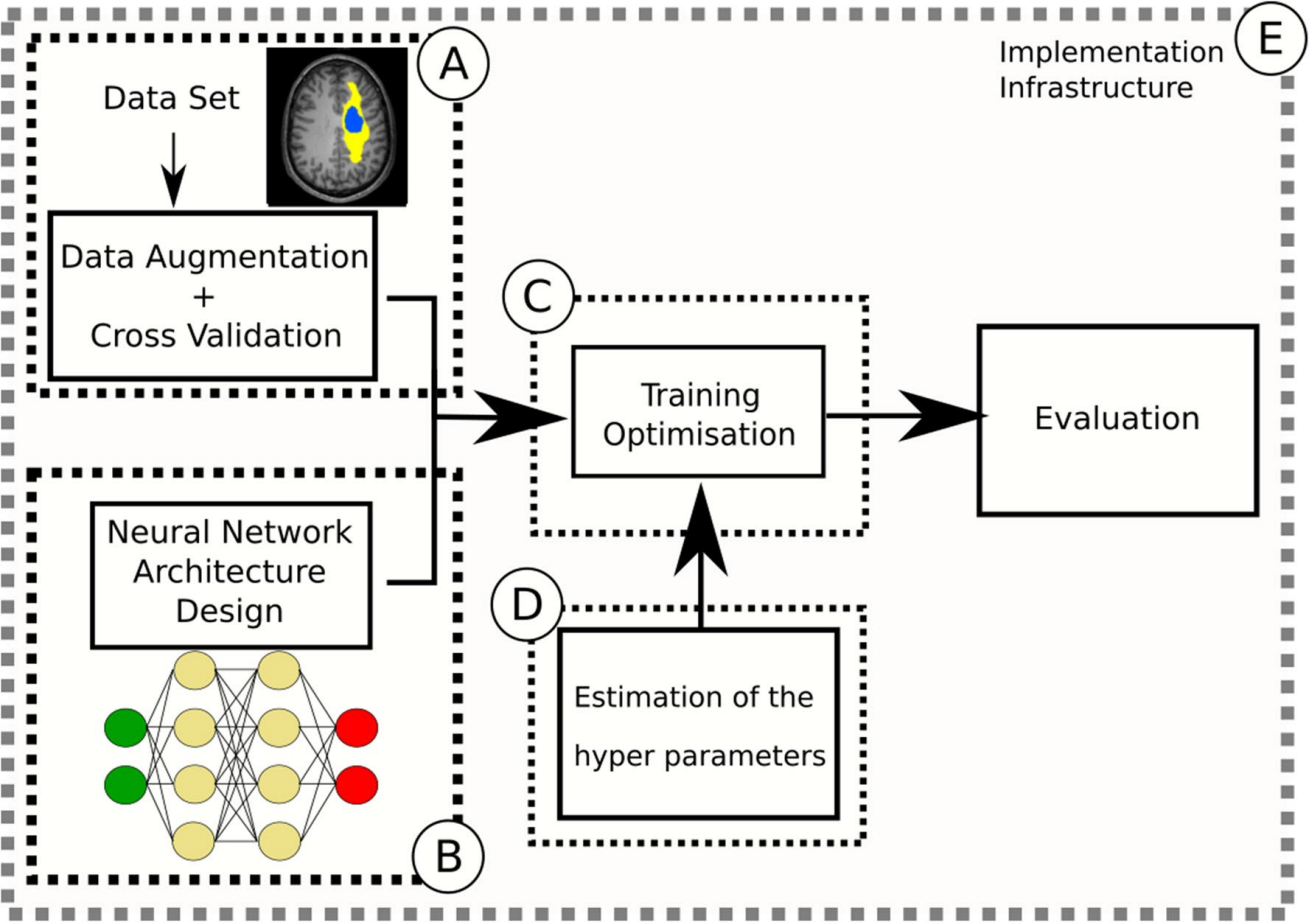
The different steps of a DL framework are displayed in solid-line boxes: the steps related to the dataset (the data augmentation and cross-validation strategies, DL architecture design, training step (with the optimization procedure), and estimation of the hyperparameters of the optimization) and the evaluation system. The different sources of variability are highlighted in dashed-line boxes: the variability linked to (**A**) the dataset, (**B**) the DL architecture, (**C**) the optimization procedure, (**D**) the hyper parameter estimation for the optimization and (**E**) the implementation and infrastructure.

In the next sections, five different kinds of variability are presented. The DL framework and its related sources of variability are displayed in Fig. 1.

#### Variability in the dataset

To infer a segmentation with a DL model (and more globally, a supervised machine learning model), the classic method consists in splitting the data sets into three parts. The first part corresponds to the “training set” for estimating the parameters of the model: it is composed of the raw data and corresponding labels. Based on the raw data, the DL algorithm infers some results that are compared to the labels. The DL parameters are then optimized to minimize the error between the results and labels. The second part is the “validation set”. It is more specific to the DL community. It estimates the unbiased error of the trained DL model. It permits the training of the DL to be stopped to avoid overfitting. It is not mandatory and is usually used in practice when the dataset has enough samples. Finally, the last part, called the “testing dataset”, provides an unbiased evaluation of the final model of the DL algorithm. The proportions of the different parts depend on the initial number of samples and can significantly affect the expected degree of generalization. Let us consider a trivial example, where only one sample is chosen for the testing set; the evaluation of the DL depends greatly on the selected sample. In the same way, selecting few samples for the training set leads the model to perfectly learn the training data.

To avoid bias in the data selection, strategies called “cross-validation” are performed. These strategies consist in dividing the dataset into several folds, then assigning these folds to the training, validation and testing sets. At the end of the DL model estimation and evaluation processes, the folds are reassigned for novel estimations and so on. The cross-validation strategies permit one to address variability in the data.

The number of parameters to estimate in a DL model is often larger than the number of images in the datasets. Moreover, in medical imaging segmentation, the heterogeneous appearance of the target organ (anatomical variability) or of the lesions (size, shape or position) poses a great challenge. One solution, called “data augmentation”^27^, generates new samples by applying different transformations to the dataset (e.g., rotation or flipping). In this way, unseen target organs or lesions can potentially be approximated. However, this also adds sources of variability in the general framework, since there is no consensus on which transformation to perform and the parameters of the transformation are generally randomly chosen.

#### Variability in the optimization

This section focuses specifically on the variability of optimization with an already estimated and constant set of hyperparameters. One of the main factors of complexity is the very large number of parameters of the model to be estimated. Training these parameters in DL models is very challenging. Solving the optimization problem of estimating these weights is generally an extremely difficult task with a stochastic process.

Each weight in the DL algorithm corresponds to another parameter (which can be seen as another dimension) of the cost function of the optimization. DL models often have millions of parameters, making the search space to be evaluated by the algorithm extremely high dimensional, in contrast to classic machine learning algorithms. Moreover, the addition of each new dimension dramatically increases the distance between points in this high-dimensional space. Consequently, the search space is drastically increased. More precisely, the number of possible distinct configurations of a set of parameters increases exponentially as the number of parameters increases. This is often referred to as the “curse of dimensionality” (see page 155 of Goodfellow et al.^13^).

In addition, the cost function is generally nonconvex (see page 282 of Goodfellow et al.^13^). These facts lead to several issues: the presence of local minima and flat regions with the constraint of the high-dimensionality of the search space. The best general algorithm known for solving this problem is stochastic gradient descent (SGD) (see chapter 5.9 of the book^13^), where the model weights are updated at each iteration using the backpropagation-of-error algorithm. However, there is no guarantee that the DL estimation will converge to a good solution (or even a good local optimum), that the convergence will be fast, or that convergence will even occur at all^28^.

Nevertheless, recent work may suggest that local minima and flat regions may be less challenging than previously believed^29–31^. From Choromanska et al.^29^, it appears that almost all local minima have very similar function values to the global optimum, and hence, finding a local minimum is sufficient. These last results have been obtained for classification tasks. Furthermore, the important convolutional step of segmentation is not considered in Choromanska et al.^29^ or Dauphin et al.^30^.

To the best of our knowledge, only one conference article^32^ addresses this issue of stochastic optimization uncertainties in medical imaging segmentation with DL. The authors show that DL models estimated several times with the same data show differences, but the results obtained on the evaluated metrics are not significantly different.

#### Variability in the hyperparameters

The hyperparameters correspond to the global settings of an algorithm. In machine learning, each parameter impacts the results differently^33^. Several hyperparameters must be fitted before the training of the DL model, for example, the learning rate for optimization and the dropout percentage for regularization^13^.

There are different ways to set them. First, manual configuration is considered. This strategy limits the exploration space, but the computation time is relatively short compared to those of other methods since only a rough approximation of the best hyperparameters is expected. The second kind of strategy is based on automatic space exploration. The classic method, called “grid search”, tests every combination of hyperparameters. It will find the best set of hyperparameters, but the computational cost increases greatly with the number of hyperparameters. Another strategy, called “random search”, randomly samples the set of hyperparameters to be evaluated. This method generally cannot reach the optimum values, but approximates them in fewer iterations than grid search.

A new strategy^34^, called “Bayesian optimization”, automatically infers a new combination of hyperparameters based on previous evaluations. In this case, the space exploration is intermediate and is driven by experience. The cost of exploration is lower than that in a grid or random search.

#### Variability in the DL architecture

Here, only the number of nodes, the number of layers, the kinds of layers (for example, convolutional, pooling, or dense) and the connections among the layers are considered in the architecture. Even with these four parameters, the number of available architectures is infinite.

In practice, only three strategies are chosen for the selection of the architecture. The first one consists in selecting a well-known DL model that has already proved its performance in previous work^15^, such as U-Net^17^ for image segmentation. This method is considered more often in clinical application fields. This method is not expected to provide the best architecture for a specific problem.

Another strategy consists in manually handcrafting the DL architecture. This leads to a plethora of architectures^15^. However, it does not guarantee the best architecture, and modifications of the tested architecture are generally not considered. The final strategy, also called “network architecture search”, is to automatically create a DL architecture through optimization for a specific task^35^. The drawback of approximating the best architecture is a very high cost in time and resources. For instance, the network architecture search proposed in^36^ tested 20,000 architectures in 4 days with 500 graphics processing units (GPUs).

The estimation of the minimal network architecture needed to achieve a certain segmentation accuracy on a given dataset can enable variability in the DL architecture to be avoided. However, as discussed in the review^37^, this topic remains a challenge.

#### Variability in the middleware and the infrastructure

The last section focuses on algorithms relevant to DL. In this section, the possible variability due to the middleware and the infrastructure is considered. There are many toolboxes to implement a DL framework. To the best of our knowledge, no publication has addressed the problem of reproducibility in DL with regard to the middleware. Different implementations are compared, for example, by programming language, in terms of their capacity to use a GPU. A review of different implementations and their characteristics can be found in^38^.

The learning phase in DL can be a very long process, considering the complexity of the architecture of the DL and the dataset size. As previously explained, the search for hyperparameters can also be prohibitive. To improve the processing time, several solutions based on the infrastructure are considered. Different kinds of infrastructures^39^ can be used, such as a central processing unit (CPU), GPU, or tensor processing unit (TPU). However, some technical characteristics such as memory precision for different memory sizes can affect the accuracy of the results^40^. Another example, the numerical operations performed on the GPU, can be nondeterministic, leading to nonreproducibility in the results^41^.

Another possibility for accelerating the processing time is choosing a parallel or distributed DL model. These techniques come with their own different methods that potentially impact the reproducibility of the outcome. For an overview of the parallel and distributed models and their own challenges, the interested reader can refer to^42, 43^.

## Methods

In this section, we first introduce how the literature review was performed, and then, we briefly describe the different metrics.

### Literature review

There is no standard for the reproducibility or evaluation of DL in medical image segmentation. The aim of this review is to reflect common practices for DL in medical image segmentation. To fulfill this expectation, this review focuses on three goals: (1) to inspect how the methods are described to enable work to be reproduced, (2) to present the variety of methodology and highlight the variability among DL frameworks and (3) to outline the kinds of evaluations used in DL.

To observe the variability of the methodology and evaluations in the literature, we focus on the 23 articles presented in the review article^15^ in the specific section “Tissue/anatomy/lesion/tumor segmentation”. This review article was chosen because it was the most relevant found on Google Scholar (with the mandatory keywords ’medical image segmentation neural network’ and at least one keyword in ’review survey’) among more than 2300 hits on Google Scholar (in December 2019). All the considered articles propose recent strategies: the oldest one was published in 2014^44^ and the mean year of publication is 2016. Moreover, the mean number of citations on Google Scholar (in December 2019) is $232.3 \pm 308.2$ (median = 97, min = 20, max = 1074).

To obtain a more recent overview, we select 3 reviews of medical image segmentation methods^37, 45, 46^. We focus specifically on how the problem of variability and reproducibility is addressed in the scientific literature.

We focus on the possible variability introduced by the data itself, by the optimization strategy and associated hyperparameters, by the middleware and the infrastructure, and by the evaluation measure. For all the inspected parameters or evaluations, we determine the presence of the terms and their potential values. This consideration is important for being able to reproduce the different works. When a framework is described, we determine whether the correct terms are used appropriately. To highlight this phenomenon, we consider the kind of algorithm used in the optimization strategy.

For the data variability, we consider whether the DL algorithm is tested on several datasets, whether they are public or private, the number of datapoints available, whether data augmentation has been performed, the proportion of training, validation and testing sets and the possible application of a cross-validation method. For the optimization, we examine whether different parameters are recorded (the optimization strategy, learning rate, batch size, and presence of dropout regularization). We also investigate whether the hyperparameters of the optimization are hand-crafted or automatically optimized (and whether this information is available). For the middleware and infrastructure considerations, we report whether these details are provided. Special attention is also paid to the implementation of the DL model and the processing unit considered. We also determine whether the calculations are performed on a distributed system, which can be a large source of variability itself. For the evaluation, we consider the number and kinds of measures, and whether the variability of the results is described (the presence of standard deviations).

### Metric evaluation

The evaluation of the different estimations of DL models is assessed with the DC, the true positive rate (TPR), also called the sensitivity (Sens.), the true negative rate (TNR), also called the specificity (Spef.), and the average volume distance (AVD) (linked with the Hausdorff distance). We chose these metrics because they often appeared in the articles of the literature review. The different metrics are described in Table 1^19^. We consider various metrics, since each metric has its drawbacks, and evaluate only a part of the segmentation problem^19, 26^. Readers interested in additional metrics and the interactions among them can read the study of Taha et al.^19^.

**Table 1:** Segmentation metrics

Metric	Equation	Range	Meaning
Dice coefficient (DC)	$\frac{2 \times |Mask ~ \cap ~Ground ~ Truth|}{|Mask| + |~Ground ~ Truth|}$	0–1	Spatial overlap between masks
True positive rate (TPR)	$\frac{TP}{TP + FN}$	0–1	Sensitivity
True negative rate (TNR)	$\frac{TN}{TN + FP}$	0–1	Specificity
Average volume distance (AVD)	$max\Big (d_H(Mask, Ground ~ Truth), d_H(Ground ~ Truth, Mask)\Big )$	$\ge 0$	Precision

*Mask* segmentation mask, *Ground Truth* ground-truth mask, *TP* true positives, voxels that are correctly segmented as the region of interest, *TN* true negatives, voxels that are correctly segmented as the background, *FP* false positives, voxels that are incorrectly segmented as the region of interest, *FN* false negatives, voxels that are incorrectly segmented as the background. $d_H$ corresponds to the directed average Hausdorff metric, defined as $d_H(A,B) = \frac{1}{N} \sum _{a \in A} \min _{b \in B} ||a - b ||$, where *N* is the number of pixels or voxels considered.

## Synthesis of the literature review

The main results are displayed in Tables 2, 3, 4 and 5. Table 2 focuses on the data variability. Table 3 focuses on the evaluation procedure. Table 4 presents the optimization strategies. Table 5 considers the middleware and the infrastructure. We are interested in the following three main points: (1) whether the DL strategy is correctly described as enabling the work to be reproduced, (2) whether the variability of the different parts of the DL framework are considered, and (3) how the evaluation is performed and the results are reported.

### Description of the DL strategy

In this section, we focus not on the fact that some methods are performed and some are not, but on whether the methods are clearly described. It can be seen that a method may have been applied without any mention in the text.

**Figure 2:**
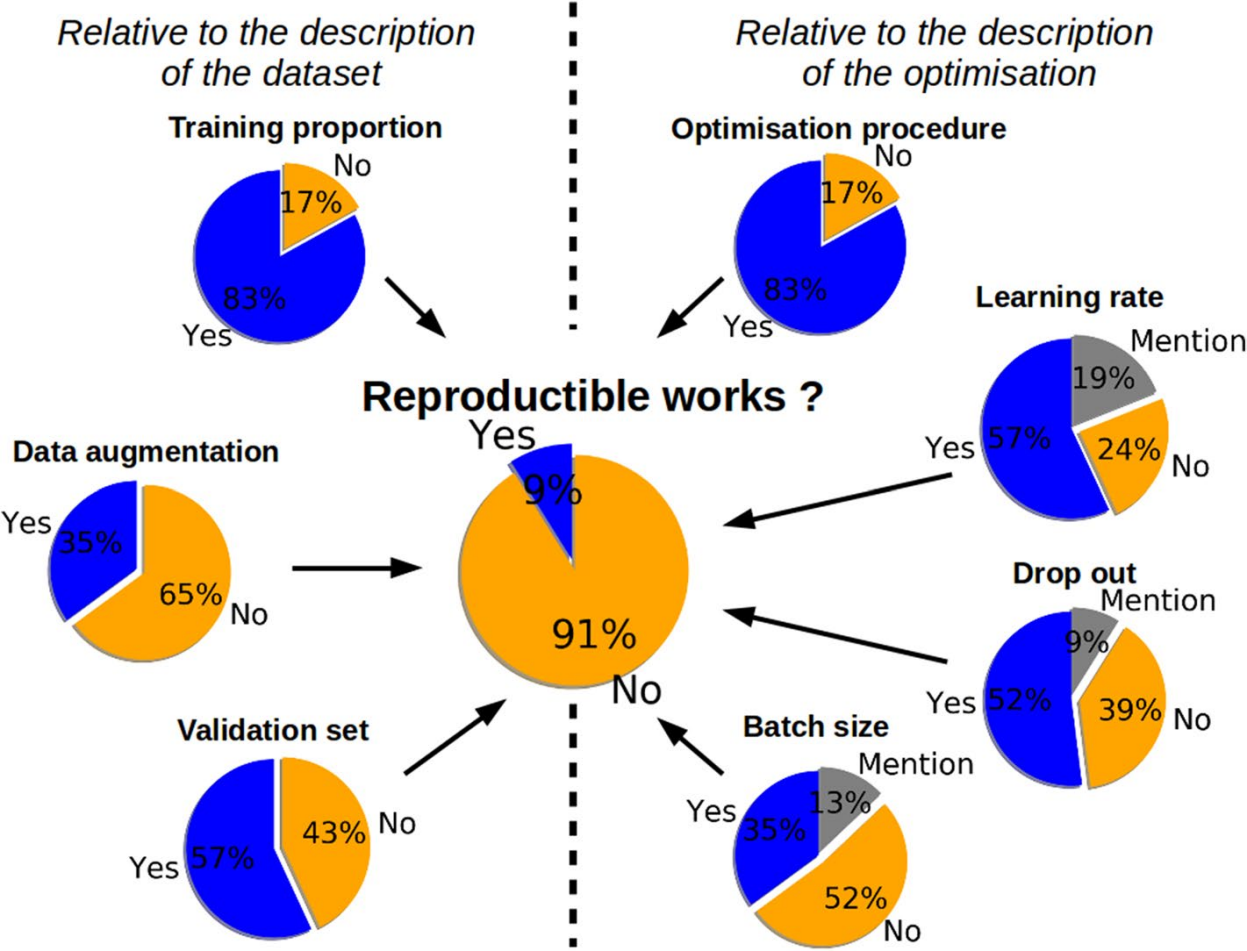
The left side, resp. the right, of the figure is relevant to the description of the dataset, resp. the optimization. The description of the training proportion is present in 83% of the selected articles. The terms of data augmentation, resp. the validation set, are described in 35%, respectively 57%, of the selected articles. For the optimization procedure, the name of the optimization algorithm is missing in 17% of the selected articles. Regarding the hyperparameter learning rate, dropout and batch size, their values are available in only 57%, 52% and 35% of the articles, respectively. These coefficients are mentioned in the text without any values in 19%, 9% and 13% of the articles, respectively. In the end, only 9% of the evaluated articles have enough information to be reproducible.

The main findings are as follows: only two articles^47, 48^ (9% of the articles) sufficiently describe the hyperparameters and the dataset to enable the work to be reproduced. One study^49^ has just one hyperparameter missing (the batch size) in the text, but the source code is available with this information included. Here, we focus on descriptions relative to the dataset and to the optimization stage. These results are detailed in Fig. 2. The left side of the figure is relevant to the description of the dataset (the training proportion, the data augmentation and the validation set) and the right side to the description of the optimization (the optimization procedure, the learning rate, the dropout procedure and the batch size). Some criteria are described well, such as the training proportion (83% of the considered articles) or the optimization procedure (83% of the selected articles). However, some characteristics are less available, such as the procedure of data augmentation (only 35% of the articles). To obtain a reproducible study, all these characteristics must be described. Only 9% of the selected articles provide sufficient information to be reproducible.

In Table 2, the dataset management method is described. All the selected articles correctly present the dataset and the number of samples. 17% of the articles do not explain the training proportion used to estimate the parameters. Only 57% of the selected articles clearly state whether they used a validation set, and 35% whether they performed data augmentation.

Table 4 focuses on the hyperparameters of the optimization process. 17% do not explain the optimization procedure at all. One^44^ cites a generic name (GDM, for gradient-based method) without any explanation. The learning rate parameter is generally present with its initial values (or range of values). Four articles do not mention the values of the parameters. For the specific AdaDelta optimization used in^50–52^, there is no learning rate. However, some coefficients need to be specified, such as the sensitivity ratio. Only one article^52^ of the three mentions this coefficient. More than half of the selected studies (52%) do not mention the batch size, and only 35% of all the articles specify its value. The dropout method, which is more relevant to regularization, is present in 61% of the selected articles (only 43% specify the dropout ratio). 43% of the selected articles state that they perform stochastic gradient descent (SGD). However, in a strict sense, SGD is a generic term, and 90% of the selected articles use SGD with momentum. Moreover, SGD is generally confused with mini-batch GDMs^53^ which is the case for 70% of the selected articles, which use the term batch size simultaneously with the term SGD.

In Table 5, it can be seen that 35% of the selected articles do not describe the toolbox for the implementation of the DL models. 26% of the selected articles do not provide the kind of infrastructure. Supposing that a correct description of a GPU needs at least the name of the constructor, the class and the memory size, only 30% have this information. It can also be observed in Table 5 that there is no convention for reporting the infrastructure.

The best way to reproduce an algorithm and to explore the hyperparameters or the architecture of a DL model is to have access to the source code. In Table 5, we observe that only 17% of the articles release the source code. These articles^47–49^ are the same as those that provide an exhaustive description of the framework for reproducibility.

### Variability in DL frameworks

**Figure 3:**
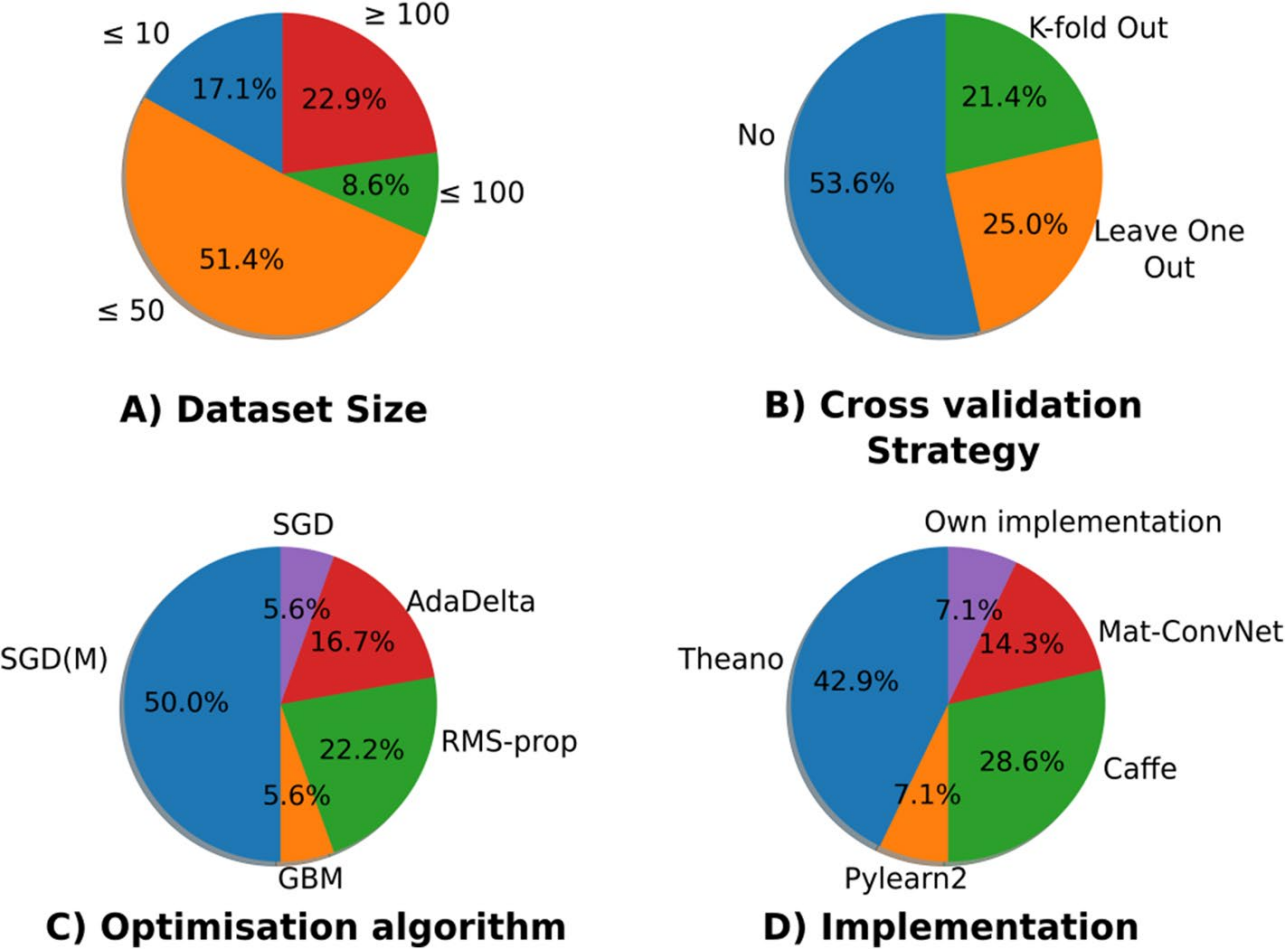
Four different sources of variability. (**A**) There is a large variability in the dataset size. 68.5% of the numbers of samples in the dataset are less than or equal to 50. (**B**) In general, no cross-validation strategy is considered (more than 50% of the articles). (**C**) There are 5 different optimization algorithms introduced in the different articles. The main one is SGD based on momentum (SGM(M)). The gradient-based method (GBM) and stochastic gradient descent (SGD) are only general terms. (**D**) There are 5 different implementations of DL frameworks. Even the Theano implementation is used in 42.9% of the considered articles, and there is no consensus among the implementations.

In the selected published articles, we are interested in the variability in the dataset, the optimization, the hyperparameters, the architecture of the DL framework, the implementation and the infrastructure. The main results in the next section are illustrated in Fig. 3. The figure is separated into four parts describing the variability of the dataset size, cross-validation strategies, optimization algorithms and implementation. The main conclusion is that there is no consensus on these topics. The rest of the results are discussed in detail in the subsequent section.

#### Variability in the dataset

In Table 3, the results are focused on data variability. More than half of the methods are evaluated on more than one dataset and with publicly available datasets (in general, provided by data challenges such as BRATS^54^.

30% of the articles only test their algorithms on private datasets.

Only 6 datasets have more than 100 samples, and in these 6 datasets, 4 come from the same public source, BRATS. This highlights the difficulty of obtaining large datasets. Consequently, data augmentation is important for medical image segmentation. Since the segmentation of a voxel can be performed locally, data augmentation based on patches can be considered. However, 13% of the articles do not clearly describe whether there is data augmentation or whether the patch strategy is considered, or how many patches are selected. The training proportion and the CV strategies permit avoiding or limiting bias relative to the chosen dataset. 52% of the articles do not use any CV strategies.

#### Variability in the optimization

One article^47^ presents an original strategy for managing the intrinsic variability in the optimization stage of the DL: the results of 3 DL models are merged, leading to better results than one alone. The other 22 articles do not discuss this notion.

#### Variability in the hyperparameters

We can observe in Table 4 that only one article,^55^, clearly explains the tuning of the hyperparameters with a grid search strategy. Another article,^56^, claims to automatically tune the hyperparameters without any explanation. In the articles considered in Table 4, there are three main strategies: SGD with momentum, RMS-prop and AdaDelta. One of the main hyperparameters is the learning rate, which varies greatly, from $10^{-2}$ to $10^{-4}$. Two articles^48, 57^ consider a range of values. At shown in Table 2, the training proportion, which can be viewed as a hyperparameter, has a wide variability (from 20% to 95% of the dataset). It is generally selected according to the size of the dataset. These results highlight the variability in the choice of hyperparameters for data management and optimization.

#### Variability in the architecture of DL frameworks

In Table 2, we can see that the main strategy is to use a convolutional neural network (CNN) or recurrent neural network (RNN) architecture (91% of the methods) for segmentation (these architectures are types of DL models^14^). Two articles^51, 58^ test several different DL architectures in their frameworks (5 for^51^ and 2 for^58^).

Only one article^55^ performed a grid search algorithm to determine the structure of the architecture (based on the kernel and max-pooling size for each layer and on the number of layers).

#### Variability in middleware and infrastructure

In Table 5, we can see that several implementations are considered. More precisely, four different toolboxes (Theano^59^, Mat-ConvNet^60^, Caffe^61^ and Pylearn2^62^) are referenced in the articles. Only one in-house implementation was used^52^.

In 13% of all the articles, a high-level API (Keras^63^ or Lasagne^64^) is deployed in addition to these toolboxes.

All the articles describing the infrastructure performed their algorithms on a GPU. No articles referred to a distributed system for the implementation of the DL algorithm.

### Evaluation of the variability

**Figure 4:**
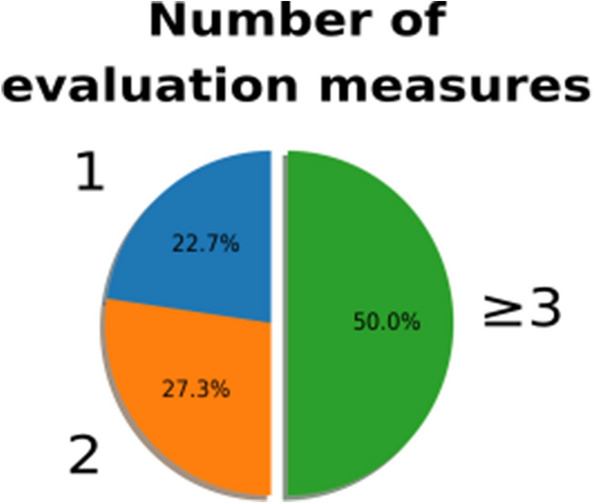
The number of evaluation measures used in each article. Note that the number of measures required to correctly evaluate a segmentation result is 3.

Almost half of the articles consider fewer than 3 metrics, which is the number recommended by Udupa et al.^26^ (see Table 3 and Fig. 4). In a quarter of the articles, no variability relative to the metrics (such as the standard deviation) is provided. In some of the cases, this can be explained by the context of the data challenge platform for evaluation. In most articles, the variability is displayed with a boxplot. Only two articles report the complete results for each participant^65, 66^.

For the evaluation metrics, the DC is considered in all articles. There is a large variability in the other metrics, since 22 different names of metrics can be found. Some of them are the same even if the names are different, such as the true positive rate, recall and sensitivity.

### Reproducibility in the literature reviews

To evaluate the impact of reproducibility in DL for image segmentation after 2017, we consider the 3 reviews^37, 45, 46^. All the reviews highlight the problem of correctly comparing different methods. To address this issue, the reviews suggest testing the DL frameworks on public datasets through challenges and providing the code publicly. Moreover, the study^45^ suggests that the difficulty of comparing the frameworks comes from the numerous available metrics used to evaluate segmentation. Furthermore, the study^37^ highlights the problem of reproducibility due to the lack of a correct description of the frameworks. Finally, all the reviews consider reproducibility as a challenge.

However, none of them raises the question of the intrinsic variability of DL frameworks. They do not refer to multiple metrics to correctly evaluate segmentation or discuss the cross-validation aspect. For the reported results in these reviews^37, 45^, no variability measure is provided, such as the standard deviation.

**Table 2:** The training size, the kind of data augmentation (DA), the presence of the DA term and the validation set (VS) term, the training size proportion and the cross-validation (CV) strategy in each article.

Article	Training size	DA	DA term	VS term	Training proportion	CV strategy
Guo (2014)^44^	$\le 50$	Patches	No	No	Not clearly detailed	LOO
de Brebisson (2015)^67^	$\le 50$	Patches	No	Yes	43%	No
Choi (2016)^68^	$\le 50$	Patches	No	Yes	75%	No
Stollenga (2015)^69^	$\le 50$	Patches	Yes	No	50%, 25%	No
Zhang (2015)^65^	$\le 10$	Patches	No	Yes	87.50%	LOO
Andermatt (2016)^50^	$\le 10$	Yes	Yes	No	25%	No
Bao (2016)^70^	$\le 10$, $\le 50$	Patches	No	No	50%, 50%	No
Birenbaum (2016)^51^	$\le 10$	Patches	Yes	Yes	80%	LOO
Brosch (2016)^52^	$\le 50$, $\le 50$, $\ge 100$	Not described	No	Yes	46%, 95%, 80%	No/LOO/No
Chen (2016a)^12^	$\le 10$	Not described	No	No	25%	LOO
Ghafoorian (2016b)	$\ge 100$	Patches	No	Yes	90%	No
Ghafoorian (2016a)	$\ge 100$	Patches	No	Yes	89%	No
Havaei (2016b)^71^	$\le 50$, $\ge 100$, $\ge 100$	Not described	No	Yes	70%	No
Havaei (2016a)^55^	$\le 50$, $\ge 100$	Patches	Yes	Yes	46%, 84%	No/7 FO
Kamnitsas (2017)^47^	$\le 100$, $\ge 100$, $\le 50$	Patches	Yes	Yes	80%, 72%, 44%	5 FO
Kleesiek (2016)^72^	$\ge 100,\le 100$	Patches	Yes	No	50%, 50%	2 FO/3 FO
Mansoor (2016)^58^	$\ge 100$	Patches	No	No	Not clearly detailed	Not clearly detailed
Milletari (2016a)^57^	$\le 100$, $\le 50$	Patches	No	Yes	82%, 33%	No
Moeskops (2016a)^56^	$\le 50$, $\le 50$, $\le 50$	Patches	No	No	20%; 25% ; 33%	LOO/No/No
Nie (2016b)^66^	$\le 10$	Patches	No	No	Not clearly detailed	LOO
Pereira (2016)^48^	$\le 50$	Patches	Yes	Yes	46%, 84%	No
Shakeri (2016)^49^	$\le 50$, $\le 50$	Patches	Yes	Yes	66% , 50%	3 FO/2 FO
Zhao (2016)^73^	$\le 50$	Patches	No	No	Not clearly detailed	Not clearly detailed

The CV method can be leave one out (LOO) or k fold out (k FO). For example, the article by Kamnitsas et al.,^47^, presents 3 datasets with training sizes $\le 100$, $\ge 100$ and $\le 50$. The data augmentation is based on a patch strategy (the authors referred to it in the article). They also explicitly described whether they used a validation set. The training proportions of the 3 datasets are 80%, 72% and 44%. Finally, the authors used 5 fold out for the CV strategy.

**Table 3:** The different DL models, kinds of datasets (number of datasets, denoted as Nb DS, the kind of dataset (public or private) and the kind of evaluation (type, number and variability of the measures).

Article	DL architecture	Nb DS	Dataset type	Type of Meas.	Nb of Meas.	Var. of Meas.
Guo (2014)^44^	SAE	1	Private	DC	1	Values
de Brebisson (2015)^67^	CNN	1	Public	DC	1	No
Choi (2016)^68^	CNN	2	Public	DC, P, R	3	Values, graph
Stollenga (2015)^69^	RNN	2	Public	DC, MHD, AVD	3	No
Zhang (2015)^65^	CNN	1	Private	DC, MHD	2	Values, graph *
Andermatt (2016)^50^	RNN	1	Public	DC, MHD, AVD	3	No
Bao (2016)^70^	CNN	2	Public	DC, VD, SD, TPR, FPR	1	No
Birenbaum (2016)^51^	CNN	1	Public	DC,Score	2	No
Brosch (2016)^52^	CNN	3	2 Public & private	DC, AVD, LTPR, LFPR	4	Graph
Chen (2016a)^12^	CNN	1	Public	DC, MHD, AVD	3	No
Ghafoorian (2016b)	CNN	1	Private	DC, AUC	2	Graph
Ghafoorian (2016a)	CNN	1	Private	DC, AUC	2	Graph
Havaei (2016b)^71^	CNN	3	Public	DC,VD,SD,TPR,FPR	5	No
Havaei (2016a)^55^	CNN	2	Public	DC,Sens.,Spe	3	Graph
Kamnitsas (2017)^47^	CNN	3	Private & 2 public	DC, P, Sens, ASSD, HD	5	Values, graph
Kleesiek (2016)^72^	CNN	4	3 Public & 1 private	DC,Sens.,Spe	3	Values, graph
Mansoor (2016)^58^	SAE	1	Private	DC, ALSD	2	Values, graph
Milletari (2016a)^57^	CNN	2	Private	DC, MDEC, FR	3	Graph
Moeskops (2016a)^56^	CNN	3	Public	DC, MSD	2	Values, graph
Nie (2016b)^66^	CNN	1	Private	DC	1	Values *
Pereira (2016)^48^	CNN	2	Public	DC, PPV, Sens	3	Graph
Shakeri (2016)^49^	CNN	2	Public & private	DC, HD, CMD	3	Graph
Zhao (2016)^73^	CNN	1	Public	DC	1	Graph

For the types of measures, *DC* Dice Coefficient, *P* Prediction, *R* Recall, *MHD* modified Hausdorff distance, *AVD* average volume distance, *TPR* true positive rate, *FPR* false positive rate, *AUC* area under the curve, *Sens.* Sensitivity, and *Spe.* Specificity. The variability of a measure corresponds to the presence of the standard deviation value or a display in a graph. The (*) means that the values for all subjects are reported. For example, the article by Kamnitsas et al.,^47^, is based on a CNN. Their models are evaluated on 3 datasets, where one is private and two public. To evaluate their segmentations, they used the DC, P., Sens., ASSD and HD metrics (5 different metrics). The variability of the measures is displayed in a graph, and the corresponding values are reported in the text.

**Table 4:** The kind of optimization, whether the hyperparameters (HPs) are handcrafted, the learning rate (the value (V.) and the presence (P.) of the term), the batch size (the value (V.) and the presence (P.) of the term), the dropout regularization (the value (V.) and the presence (P.) of the term) and whether the code is open source.

Article	Optimization	HP handcrafted	Learning rate (V./P.)	Batch size (V./P.)	Dropout (V./P.)
Guo (2014)^44^	GBM	Yes	No/no	No	No
de Brebisson (2015)^67^	SGD (M)	Yes	Yes (0.05)/yes	Yes/yes	No
Choi (2016)^68^	SGD (M)	Yes	Yes (0.001)/yes	No/yes	Yes/yes
Stollenga (2015)^69^	RMS-prop	Yes	Yes (0.01)/yes	No	Yes/yes
Zhang (2015)^65^	SGD (M)	Yes	Yes (0.0001)/yes	No	Yes/yes
Andermatt (2016)^50^	AdaDelta	Yes	omit	No	Yes/yes
Bao (2016)^70^	Not described	Yes	No	No	No
Birenbaum (2016)^51^	AdaDelta	Yes **	omit	No	Yes/yes
Brosch (2016)^52^	AdaDelta	Yes	Sensitivity ratio Yes/yes	No	No
Chen (2016a)^12^	Not described	Yes	No	No	No
Ghafoorian (2016b)	RMS-prop	Yes	No/yes	Yes/yes	Yes/yes
Ghafoorian (2016a)	RMS-prop	Yes	No/yes	Yes/yes	Yes/yes
Havaei (2016b)^71^	SGD (M)	Yes	Yes (0.001)/yes	No	No/yes
Havaei (2016a)^55^	SGD (M)	No (Grid Search)	Yes(0.005)/yes	No/yes	Yes/yes
Kamnitsas (2017)^47^	RMS-prop	Yes	Yes(0.0001)/yes	Yes/yes	Yes/yes
Kleesiek (2016)^72^	SGD	Yes	Yes(0.00001)/yes	Yes/yes	No
Mansoor (2016)^58^	SGD (M)	Yes **	No	Yes/yes	No
Milletari (2016a)^57^	SGD (M)	Yes	Yes (range values)/yes	Yes/yes	Yes/yes
Moeskops (2016a)^56^	RMS-prop	No (not explained)	No/yes	No/yes	No/yes
Nie (2016b)^66^	Not described	Yes	No/yes	No	No
Pereira (2016)^48^	SGD (M)	Yes	Yes (range values)/yes	Yes/yes	Yes/yes
Shakeri (2016)^49^	SGD (M)	Yes	Yes(0.01)/yes	No	Yes/yes
Zhao (2016)^73^	Not described	Yes	No	No	No

The (M) in the optimization column signifies that the momentum algorithm is performed. The ** in the HP handcrafted column means that several DL architectures are tested. For example, the article by Kamnitsas et al.,^47^, used an RMS-prop strategy for optimization. The different hyperparameters are handcrafted. The learning rate, the batch size and the dropout are mentioned in the text, and their corresponding values are given.

**Table 5:** In the second column, the different implementations are described (Theano $^{1}$, Mat-ConvNet $^{2}$, Caffe $^{3}$, Keras $^{4}$, Pylearn2 $^{5}$ and Lasagne $^{6}$).

Articles	Implementation	Infrastructure	Open source
Guo (2014)^44^	Not described	Not described	No
de Brebisson (2015)^67^	Theano	NVIDIA Tesla K40 GPU-12GB	No
Choi (2016)^68^	Mat-ConvNet	GPU (GTX TITAN)	No
Stollenga (2015)^69^	Not described	NVIDIA GTX TITAN X GPU-12GB	No
Zhang (2015)^65^	Not described	Tesla K20c GPU	No
Andermatt (2016)^50^	Caffe	NVIDIA GTX Titan X GPU-12GB	No
Bao (2016)^70^	Not described	Not described	No
Birenbaum (2016)^51^	Keras + Theano	NVIDIA GeForce GTX 980 Ti GPU	No
Brosch (2016)^52^	Own implementation	GeForce GTX 780	No
Chen (2016a)^12^	Caffe	NVIDIA TITAN X GPU	Yes (*)
Ghafoorian (2016b)^7^	Theano	Not described	No
Ghafoorian (2016a)^7^	Not described	Titan X card	No
Havaei (2016b)^71^	Keras	Nvidia TitanX GPU	No
Havaei (2016a)^55^	Pylearn2	NVIDIA Titan black card.	No
Kamnitsas (2017)^47^	Theano	NVIDIA GTX Titan X GPU-12GB	Yes
Kleesiek (2016)^72^	Theano	NVIDIA Titan-3GB	No
Mansoor (2016)^58^	Not described	Not described	No
Milletari (2016a)^57^	Caffe	NVIDIA “‘Tesla k40” or “Titan X”-12GB	No
		Tested on Nvidia GTX 980-4GB	No
Moeskops (2016a)^56^	Not described	NVIDIA Tesla K40 GPU (**)	No
Nie (2016b)^66^	Caffe	Not described	No
Pereira (2016)^48^	Theano + Lasagne	GPU NVIDIA GeForce GTX 980	Yes
Shakeri (2016)^49^	Mat-ConvNet	Described on github	Yes
Zhao (2016)^73^	Not described	Not described	No

$^{1}$http://deeplearning.net/software/theano/.

$^{2}$http://www.vlfeat.org/matconvnet/.

$^{3}$https://caffe.berkeleyvision.org/.

$^{4}$https://keras.io/.

$^{5}$http://deeplearning.net/software/pylearn2/.

$^{6}$https://lasagne.readthedocs.io/en/latest/.

For the infrastructure details, the materials are described as they are referenced in the articles. If the global memory is reported in the article, it is noted. The last column, ’Open Source’, shows whether the source code is available. The (*) indicates that the code source is not available but a detailed prototype of the algorithm is provided. The (**) indicates that the infrastructure is detailed in the Acknowledgements section. For example, the article by Kamnitsas et al.,^47^, used the Theano implementation on an infrastructure based on an NVIDIA GTX Titan X GPU-12GB. Their code is released as open source.

## Proposals for practices conducive to reproducibility in medical image segmentation with DL

On the basis of the literature review, our recommendations focus on three main points: (1) an adequate description of the DL framework, (2) a suitable analysis of the different sources of variability in the DL framework, and (3) an efficient evaluation system for the segmentation results.

**Figure 5:**
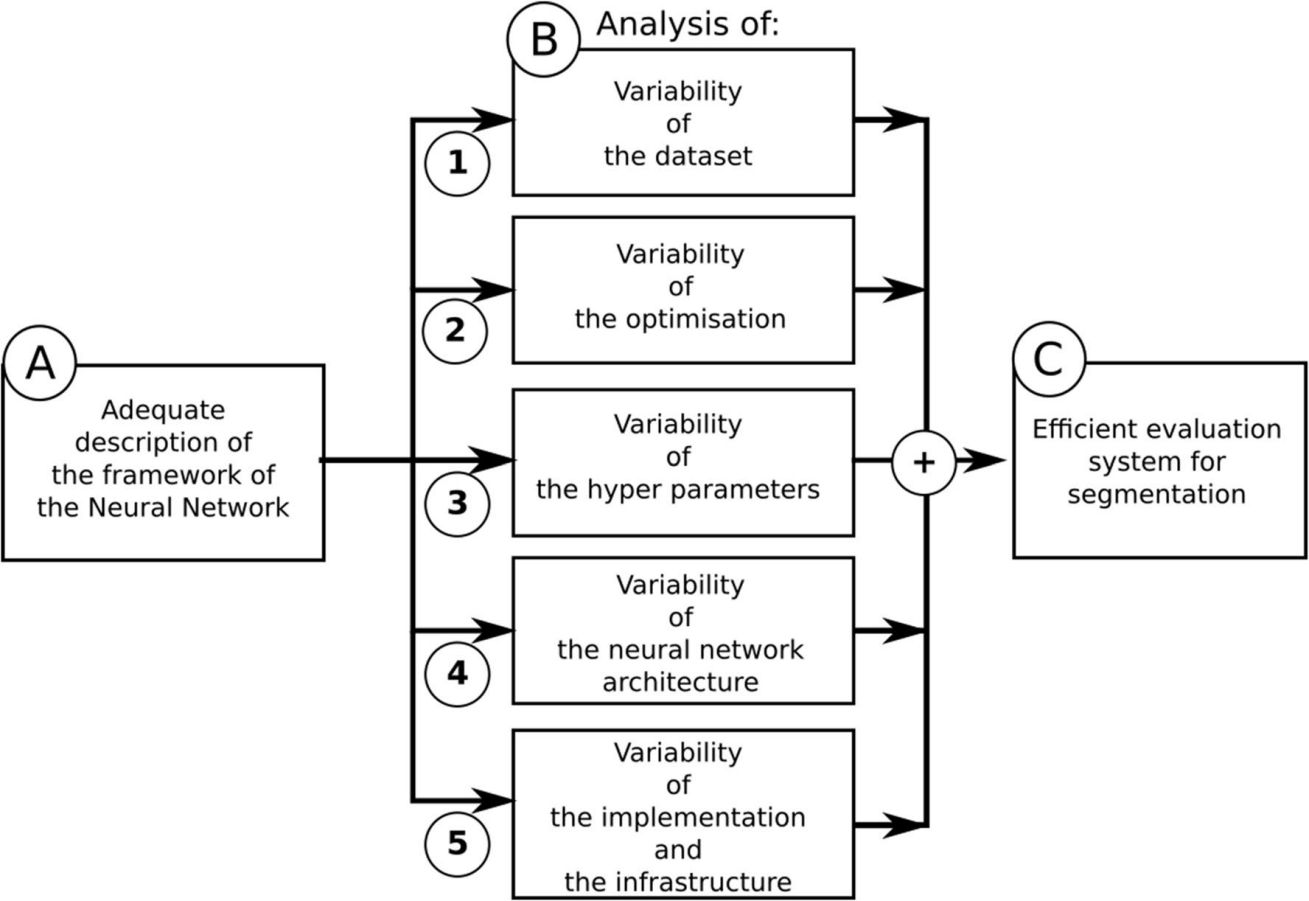
The proposals are separated into three main parts: (**A**) an adequate and complete description of the DL framework for reproducibility purposes, (**B**) an analysis of the different sources of variability, and (**C**) an efficient evaluation system for image segmentation.

The flowchart of the different proposals is displayed in Fig. 5. Even if each part is independent, there is a natural order that we follow in our recommendations.

### Recommendations for the description of the framework

First, to perform reproducible research, it is mandatory to correctly describe all the aspects of the framework, from the DL model and its related hyperparameters to the evaluation system. The initial step consists in clearly describing the algorithm and/or the model of the DL architecture. A schema of the DL architecture should be provided since the architecture is generally complex.

For the data part, several steps are mandatory:

- A complete description of the dataset is required, with the kind of acquisition (i.e., MRI or scanner), the size of the images and the total sample size. If the dataset is publicly available, a download link should be provided.
- For the preprocessing stage, the authors should explain whether some data are excluded. In the case of data augmentation, the different kinds of transformation must be described and the final number of samples should be included. For the special case of images, if the data augmentation consists in the selection of multiple patches, the characteristics and the final number of patches should be described.
- The allocation of the dataset samples into training, validation, and testing sets should be clearly described. If no validation set is created, this must be clearly stated and the choice should be explained.
- The cross-validation strategy should be described along with the number of folds considered.

For the optimization step, the chosen algorithm should be clearly referenced with its name and its corresponding publication, and the final hyperparameters, such as the learning rate or the batch size, should be provided. If several evaluations are performed, the number of trials should be given.

For the selection of the hyperparameters of the optimization process or the design of the DL architecture, the method should be explained, even if it is handcrafted. More precisely, the method and the search space of the different hyperparameters should be provided.

A description of the computing infrastructure should be given with technical specifications: at least the name of the constructor, the class of the architecture and the memory size should be provided. For the middleware, the kind of implementation should be described (an available toolbox or in-house code, and the build version). If the toolbox is public, the link to the toolbox should be available. In general, the best solution is to provide a link to the downloadable source code with all included dependencies.

Finally, for the evaluation, a clear description of the results should be given with the average metrics and their variations. If a figure is displayed, such as a boxplot, the values of the error bars should be provided.

All these recommendations have been proposed in the *Machine Learning Reproducibility Checklist*^18^. There are two main differences between their recommendations and ours. First we merge the different points by the source of variability, whereas they merge by the section of the article (Methods and Results). The second difference consists in the particular focus on the image segmentation proposed here.

### Recommendations for the analysis of variability

As shown by the literature review, sources of variability occur in each part of a DL framework: the dataset, the optimization procedure, the selection of the hyperparameters, the DL architecture, and the computational infrastructure. Each kind of variability is different and should be considered with its own tools.

#### Variability of the dataset

In practice, the available data are always a subset (or sampling) of the true distribution. This sampling effect typically introduces a bias which, in turn, results in variability in the final results.

This fact is important in medical image processing, where the number of samples is limited. A DL network learning from a particular sampling of the data can lead to overfitting. The recommended common tool is cross-validation. It must be noted that the purpose of cross-validation is different from the use of batches in optimization. The choice of the cross-validation method (the number of samples left out) should be considered with regard to the sample size (i.e., as in the leave one out or k fold out strategy). The choice of the samples to be analyzed (patches or 2D images from 3D images) can lead to strong correlations between samples.

To obtain a better sampling of the true distribution of the training dataset, data augmentation must be considered. The different transformations for the augmentation must be chosen carefully with regard to the organs studied. Furthermore, this data augmentation can enhance the accuracy by correcting a very poor diversity of the training dataset with respect to the testing dataset.

#### Variability of the optimization

For the optimization, the analysis of variability is often studied for classification purposes^29–31^. Our recommendation to manage the variability of the optimization is to perform at least several trials with the same hyperparameters on the same datasets. The observed variability should be recorded in the average score and its corresponding standard deviation.

For a deeper analysis taking account of the dataset variability, the optimization should be produced several times in a cross-validation strategy for the dataset. All the results for each fold of the dataset should be grouped. A one-way ANOVA statistical test should be performed on the different groups to test whether there is a difference or interaction over the dataset considering the optimization. If the assumptions of ANOVA are violated, a strategy to perform a nonparametric test over cross-validation has already been proposed^74^ to better estimate the residual error and to analyze the interaction between the algorithm and the learning dataset.

#### Variability of the hyperparameters

Our recommendation for the selection of the hyperparameters is to avoid handcrafted selection. Even if this selection is fast, the set of hyperparameters obtained can have a high variability since the hyperparameters can lie in a range of large variability^33^. Automatic selection by a grid search, random search or Bayesian optimization algorithm enables optimum values to be obtained that are potentially more robust. It should be noted that a *Network Architecture Search Best Practices Checklist*^75^ was written in September 2019 on this specific subject.

#### Variability of DL architectures

The main problem addressed by the evaluated articles is: how should different DL architectures be compared? The comparison should consider the variability in the dataset, the optimization and the hyperparameter selection. Our recommendation is to perform, for each evaluated DL architecture, several trials of optimization on each fold of the dataset provided by a cross-validation strategy. A two-way ANOVA can be considered to evaluate the variability of the metrics with regard to the different folds of the cross-validation and the different DL architectures. If the assumptions of the ANOVA are violated, a nonparametric test can be proposed^76^.

#### Variability of the infrastructure

In general, it is difficult to test the variability of infrastructures since their costs can be high. Our recommendations are to correctly capture the specificity of the infrastructure to avoid side effects for the reproducibility. Two important factors are the number of processing units and their characteristics (the kind of calculus unit used and the available random access memory (RAM)). The number of processor units will deeply impact the framework (distributed or non-distributed system). The RAM can affect the size of the batch during optimization. The kind of calculus unit used can lead to quantization and problematic noise calculations in the optimization.

Regarding the middleware, an automatic deployment of the operating system and the toolbox associated with the DL framework are recommended. This should be based on a complete description of the system.

In addition, distributed systems can be considered to achieve simulations in a reasonable time. To mitigate the reproducibility problem, some recommendations for the network should be made, such as the use of Infiniband (to avoid latency) or the use of a compartmentalized network (to avoid interactions with other users).

### Recommendations for the analysis of the evaluation system

In the context of image segmentation, at least three metrics should be considered^19^. Because some of them are correlated^19^, it is important to carefully choose which metric suits the scenario at hand^26^.

Even if several metrics are defined^19^ and, to the best of our knowledge, no consensus exists, we propose to at least evaluate the segmentation methods with the next three most common metrics: the DC, the TPR and the FNR. These metrics are described in the Methods section. Readers interested in image segmentation metrics can see more complex evaluations based on the recommendations of some studies^19, 26^.

## Discussion

The complexity and heterogeneity of DL frameworks are responsible for multiple kinds of variability. Because of the reproducibility crisis^21, 22^, researchers have highlighted multiple factors that induce variability in the results obtained, as well as important guidelines that must be respected in order to minimize—or at least quantify—these effects: (i) for other researchers to be able to replicate the obtained results, it is necessary to precisely describe the DL framework in use as well as its optimization procedure; (ii) potential sources of variability must be acknowledged and, when possible, evaluated in order to determine their importance. Last, it is crucial to consider the specifics of the field being researched: already-existing data processing methodologies and evaluation procedures must be properly incorporated within the DL framework—see for instance^19, 26^ for medical image segmentation. In practice, however, assessing reproducibility and variability is a rather difficult task in the context of DL frameworks.

- *Key factors are generally interdependent* For instance, the variability due to the optimization procedure not only depends on the choice of hyperparameters but also on the input data provided, i.e., the datasets. Facing such an issue, there is a need for new mathematical tools: (i) to de-correlate the overall variability and capture the individual effects associated with given parameter subsets; and (ii) to better compare the results obtained with different DL solutions.
- *Heterogeneous nature of the variability* This effect often makes it difficult to relate different sources of variability. For instance, let us consider the variability in the input data distribution on the one hand, and the variability in the optimization stochastic process on the other hand: these cannot be addressed in the same way, which in turn leads to different mathematical tools being needed to evaluate this variability.
- *Hardware/software perturbations* Typically, variability is estimated from a large number of repeated simulations, which requires powerful and/or distributed systems. These systems also induce variability, as they may differ slightly (in terms of architecture, data quantization, rounding strategies, implementation constraints, etc.).

Conversely, variability may also be seen as a blessing. For instance, merging different optimization solutions or different DL frameworks improves the segmentation^47^ and, more generally, the robustness.

Finally, there is no clear consensus on the meaning of reproducibility, robustness and generalizability^77^. The notion of reproducibility should be driven mainly by the kind of application.

## References

[1] Withey DJ, Koles ZJ (2008). A review of medical image segmentation: methods and available software. Int. J. Bioelectromagn..

[2] Sharma N, Aggarwal LM (2010). Automated medical image segmentation techniques. J. Med. Phys. Assoc. Med. Phys. India.

[3] Mezer A (2013). Quantifying the local tissue volume and composition in individual brains with magnetic resonance imaging. Nat. Med..

[4] Sharma K (2017). Automatic segmentation of kidneys using deep learning for total kidney volume quantification in autosomal dominant polycystic kidney disease. Sci. Rep..

[5] Silveira M (2009). Comparison of segmentation methods for melanoma diagnosis in dermoscopy images. IEEE J. Sel. Top. Signal Process..

[6] Chrástek R (2005). Automated segmentation of the optic nerve head for diagnosis of glaucoma. Med. Image Anal..

[7] Ghafoorian M (2017). Location sensitive deep convolutional neural networks for segmentation of white matter hyperintensities. Sci. Rep..

[8] Trebeschi S (2017). Deep learning for fully-automated localization and segmentation of rectal cancer on multiparametric mr. Sci. Rep..

[9] Fischl B (2002). Whole brain segmentation: automated labeling of neuroanatomical structures in the human brain. Neuron.

[10] Tu Z (2008). Brain anatomical structure segmentation by hybrid discriminative/generative models. IEEE Trans. Med. Imaging.

[11] Fortunati V (2013). Tissue segmentation of head and neck ct images for treatment planning: a multiatlas approach combined with intensity modeling. Med. Phys..

[12] Chen X, Xu L, Yang Y, Egger J (2016). A semi-automatic computer-aided method for surgical template design. Sci. Rep..

[13] Goodfellow I, Bengio Y, Courville A (2016). Deep Learning.

[14] LeCun Y, Bengio Y, Hinton G (2015). Deep learning. Nature.

[15] Litjens G (2017). A survey on deep learning in medical image analysis. Med. Image Anal..

[16] Long, J., Shelhamer, E. & Darrell, T. Fully convolutional networks for semantic segmentation. *Proceedings of the IEEE Conference on Computer Vision and Pattern Recognition* 3431–3440 (2015). 10.1109/TPAMI.2016.2572683 27244717

[17] Ronneberger, O., Fischer, P. & Brox, T. U-Net: convolutional networks for biomedical image segmentation. In *International Conference on Medical Image Computing and Computer-Assisted Intervention* 234–241 (Springer, 2015).

[18] Pineau, J. *et al.**Improving Reproducibility in Machine Learning Research (A Report from the Neurips 2019 Reproducibility Program)*. arXiv:2003.12206 (2020).

[19] Taha AA, Hanbury A (2015). Metrics for evaluating 3d medical image segmentation: analysis, selection, and tool. BMC Med. Imaging.

[20] National Academies of Sciences, Engineering, and Medicine (2019). Reproducibility and Replicability in Science.

[21] Baker M (2016). 1,500 scientists lift the lid on reproducibility. Nat. News.

[22] Stupple A, Singerman D, Celi LA (2019). The reproducibility crisis in the age of digital medicine. NPJ Digit. Med..

[23] Shrout PE, Fleiss JL (1979). Intraclass correlations: uses in assessing rater reliability. Psychol. Bull..

[24] Fisher RA (2006). Statistical Methods for Research Workers.

[25] Browne MW (2000). Cross-validation methods. J. Math. Psychol..

[26] Udupa JK (2006). A framework for evaluating image segmentation algorithms. Comput. Med. Imaging Graph..

[27] Shorten C, Khoshgoftaar TM (2019). A survey on image data augmentation for deep learning. J. Big Data.

[28] LeCun, Y. A., Bottou, L., Orr, G. B. & Müller, K.-R. Efficient backprop. In *Neural Networks: Tricks of the Trade*, 9–48 (Springer, 2012).

[29] Choromanska, A., Henaff, M., Mathieu, M., Arous, G. B. & LeCun, Y. The loss surfaces of multilayer networks. In *Artificial Intelligence and Statistics* 192–204 (2015).

[30] Dauphin, Y. N. *et al.* Identifying and attacking the saddle point problem in high-dimensional non-convex optimization. In *Advances in Neural Information Processing Systems* 2933–2941 (2014).

[31] Goodfellow, I. J., Vinyals, O. & Saxe, A. M. *Qualitatively Characterizing Neural Network Optimization Problems*. arXiv:1412.6544 (2014).

[32] Piantadosi, G., Marrone, S. & Sansone, C. On reproducibility of deep convolutional neural networks approaches. In *International Workshop on Reproducible Research in Pattern Recognition* 104–109 (Springer, 2018).

[33] Hutter, F., Hoos, H. & Leyton-Brown, K. An efficient approach for assessing hyperparameter importance. In *Proceedings of International Conference on Machine Learning 2014 (ICML 2014)*, 754–762 (2014).

[34] Bergstra, J., Yamins, D. & Cox, D. D. Hyperopt: a python library for optimizing the hyperparameters of machine learning algorithms. In *Proceedings of the 12th Python in Science Conference*, 13–20 (Citeseer, 2013).

[35] He, X., Zhao, K. & Chu, X. *Automl: A Survey of the State-of-the-Art* (2019). arXiv:1908.00709.

[36] Zoph, B., Vasudevan, V., Shlens, J. & Le, Q. V. Learning transferable architectures for scalable image recognition. *Proceedings of the IEEE Conference on Computer Vision and Pattern Recognition* 8697–8710 (2018).

[37] Minaee, S. *et al.**Image Segmentation Using Deep Learning: A Survey*. arXiv:2001.05566 (2020). 10.1109/TPAMI.2021.3059968 33596172

[38] Sherkhane, P. & Vora, D. Survey of deep learning software tools. In *2017 International Conference on Data Management, Analytics and Innovation (ICDMAI)* 236–238 (IEEE, 2017).

[39] Wang, Y., Wei, G. & Brooks, D. *Benchmarking TPU, GPU, and CPU Platforms for Deep Learning*. arXiv:1907.10701 (2019).

[40] Gupta, S., Agrawal, A., Gopalakrishnan, K. & Narayanan, P. Deep learning with limited numerical precision. In *International Conference on Machine Learning* 1737–1746 (2015).

[41] Nagarajan, P., Warnell, G. & Stone, P. The impact of nondeterminism on reproducibility in deep reinforcement learning. In *2nd Reproducibility in Machine Learning Workshop at ICML 2018, Stockholm, Sweden* (2018).

[42] Ben-Nun T, Hoefler T (2019). Demystifying parallel and distributed deep learning: an in-depth concurrency analysis. ACM Comput. Surv. (CSUR).

[43] Mayer, R. & Jacobsen, H.-A. *Scalable Deep Learning on Distributed Infrastructures: Challenges, Techniques and Tools*. arXiv:1903.11314 (2019).

[44] Guo, Y. *et al.* Segmenting hippocampus from infant brains by sparse patch matching with deep-learned features. In *International Conference on Medical Image Computing and Computer-Assisted Intervention* 308–315 (Springer, 2014). 10.1007/978-3-319-10470-6_39 PMC4445142 25485393

[45] Zhou T, Ruan S, Canu S (2019). A review: deep learning for medical image segmentation using multi-modality fusion. Array.

[46] Lundervold AS, Lundervold A (2019). An overview of deep learning in medical imaging focusing on MRI. Zeitschrift für Medizinische Physik.

[47] Kamnitsas K (2017). Efficient multi-scale 3d CNN with fully connected crf for accurate brain lesion segmentation. Med. Image Anal..

[48] Pereira S, Pinto A, Alves V, Silva CA (2016). Brain tumor segmentation using convolutional neural networks in MRI images. IEEE Trans. Med. Imaging.

[49] Shakeri, M. *et al.* Sub-cortical brain structure segmentation using f-CNN’s. In *2016 IEEE 13th International Symposium on Biomedical Imaging (ISBI)* 269–272 (IEEE, 2016).

[50] Andermatt, S., Pezold, S. & Cattin, P. Multi-dimensional gated recurrent units for the segmentation of biomedical 3d-data. In *Deep Learning and Data Labeling for Medical Applications* 142–151 (Springer, 2016).

[51] Birenbaum, A. & Greenspan, H. Longitudinal multiple sclerosis lesion segmentation using multi-view convolutional neural networks. In *Deep Learning and Data Labeling for Medical Applications* 58–67 (Springer, 2016).

[52] Brosch T (2016). Deep 3d convolutional encoder networks with shortcuts for multiscale feature integration applied to multiple sclerosis lesion segmentation. IEEE Trans. Med. Imaging.

[53] Karpathy, A. *Cs231n Convolutional Neural Networks for Visual Recognition*. http://cs231n.github.io/optimization-1/ (2020).

[54] Menze BH (2014). The multimodal brain tumor image segmentation benchmark (brats). IEEE Trans. Med. Imaging.

[55] Havaei M (2017). Brain tumor segmentation with deep neural networks. Med. Image Anal..

[56] Moeskops P (2016). Automatic segmentation of mr brain images with a convolutional neural network. IEEE Trans. Med. Imaging.

[57] Milletari F (2017). Hough-cnn: deep learning for segmentation of deep brain regions in MRI and ultrasound. Comput. Vis. Image Underst..

[58] Mansoor A (2016). Deep learning guided partitioned shape model for anterior visual pathway segmentation. IEEE Trans. Med. Imaging.

[59] Theano Development Team. *Theano: A Python Framework for Fast Computation of Mathematical Expressions*. **abs/1605.02688** (2016).

[60] Vedaldi, A. & Lenc, K. Matconvnet—convolutional neural networks for matlab. In *Proceeding of the ACM International Conference on Multimedia* (2015).

[61] Jia, Y. *et al.**Caffe: Convolutional Architecture for Fast Feature Embedding*. arXiv:1408.5093 (2014).

[62] Goodfellow, I. J. *et al.**Pylearn2: A Machine Learning Research Library*. arXiv:1308.4214 (2013).

[63] Gulli A, Pal S (2017). Deep Learning with Keras.

[64] Dieleman, S. *et al.* Lasagne: First release. 10.5281/zenodo.27878 (2015).

[65] Zhang W (2015). Deep convolutional neural networks for multi-modality isointense infant brain image segmentation. NeuroImage.

[66] Nie, D., Wang, L., Gao, Y. & Shen, D. Fully convolutional networks for multi-modality isointense infant brain image segmentation. In *2016 IEEE 13th international symposium on biomedical imaging (ISBI)* 1342–1345 (IEEE, 2016). 10.1109/ISBI.2016.7493515 PMC5031138 27668065

[67] de Brebisson, A. & Montana, G. Deep neural networks for anatomical brain segmentation. *Proceedings of the IEEE Conference on Computer Vision and Pattern Recognition Workshops* 20–28 (2015).

[68] Choi H, Jin KH (2016). Fast and robust segmentation of the striatum using deep convolutional neural networks. J. Neurosci. Methods.

[69] Stollenga, M. F., Byeon, W., Liwicki, M. & Schmidhuber, J. Parallel multi-dimensional lstm, with application to fast biomedical volumetric image segmentation. *Advances in neural information processing systems* 2998–3006 (2015).

[70] Bao S, Chung AC (2018). Multi-scale structured cnn with label consistency for brain MR image segmentation. Comput. Methods Biomech. Biomed. Eng. Imaging Vis..

[71] Havaei, M., Guizard, N., Chapados, N. & Bengio, Y. Hemis: hetero-modal image segmentation. In *International Conference on Medical Image Computing and Computer-Assisted Intervention* 469–477 (Springer, 2016).

[72] Kleesiek J (2016). Deep MRI brain extraction: a 3D convolutional neural network for skull stripping. NeuroImage.

[73] Zhao, L. & Jia, K. Multiscale CNNS for brain tumor segmentation and diagnosis. In *Computational and mathematical methods in medicine* 2016 (2016). 10.1155/2016/8356294 PMC4812495 27069501

[74] Piater JH, Cohen PR, Zhang X, Atighetchi M (1998). A randomized anova procedure for comparing performance curves. ICML.

[75] Lindauer, M. & Hutter, F. Best practices for scientific research on neural architecture search. arXiv:1909.02453 (2019).

[76] Demšar J (2006). Statistical comparisons of classifiers over multiple data sets. J. Mach. Learn. Res..

[77] Bollen Kenneth, J. T., Cacioppo, Kaplan, R. M., Krosnick, J. A., Olds, J. L. & Dean, H. Social, behavioral, and economic sciences perspectives on robust and reliable science. In *National Science Foundation Report* (2015).

